# Return

**Published:** 1888-01-21

**Authors:** A. C. Swinburne


### "RETURN."

"Return," we dare not as we fain
  Would cry from hearts that yearn:
Love does not bid our dead again
  Return.

  O hearts that strain and burn
As fires fast fettered burn and strain!
  Bow down, lie still, and learn.

The heart that healed all hearts of pain
  No funeral rites inurn;
Its echoes, while the stars remain,
  Return.

—*A. C. Swinburne.*

